# Outcomes of adjunctive surgery for nontuberculous mycobacterial pulmonary disease

**DOI:** 10.1186/s12890-021-01679-0

**Published:** 2021-10-06

**Authors:** Joong-Yub Kim, Samina Park, In Kyu Park, Chang Hyun Kang, Young Tae Kim, Jaemoon Koh, Jae-Joon Yim, Nakwon Kwak

**Affiliations:** 1 Division of Pulmonary and Critical Care Medicine, Department of Internal Medicine, Seoul National University Hospital, Seoul, South Korea; 2 Department of Thoracic and Cardiovascular Surgery, Seoul National University Hospital, Seoul, South Korea; 3 Department of Thoracic and Cardiovascular Surgery, Seoul National University College of Medicine, Seoul, South Korea; 4 Department of Pathology, Seoul National University Hospital, Seoul, South Korea; 5 Department of Pathology, Seoul National University College of Medicine, Seoul, South Korea; 6 Department of Internal Medicine, Seoul National University College of Medicine, Seoul, South Korea

**Keywords:** Nontuberculous mycobacteria, Surgery, Resection, Treatment outcome

## Abstract

**Background:**

Owing to the unsatisfactory results of antibiotic treatment alone, surgical resection is currently considered as adjunctive therapy in patients with nontuberculous mycobacterial pulmonary disease (NTM-PD). However, reports regarding the outcomes of surgery vary considerably by institution. Here, we investigated the surgical outcomes and risk factors associated with unfavorable outcomes after surgery.

**Methods:**

We analyzed patients with NTM-PD who underwent pulmonary resection at Seoul National University Hospital between January 1, 2006, and December 31, 2020, and assessed the types of surgical procedures, complications, and long-term outcomes. Multivariate logistic regression analysis was used to identify the risk factors associated with treatment refractoriness or recurrence after surgery.

**Results:**

Among 67 patients who underwent surgery during the study period, the most common indication for surgery was persistent culture positivity despite rigorous medical treatment (80.6%), followed by longstanding cavitary lesions or radiographic aggravation (10.4%) and massive hemoptysis (4.5%). Among 53 patients with positive mycobacterial cultures at the time of surgery, 38 (71.7%) achieved initial negative culture conversion, 9 (17.0%) of whom experienced recurrence. Nine (13.4%) patients experienced postoperative complications, which were managed without lasting morbidity and mortality. Female sex (adjusted odds ratio [aOR] 6.63; 95% confidence interval [CI] 1.04–42.4; *P* = .046), preoperative positive mycobacterial culture (aOR 5.87; 95 %CI 1.04–33.08; *P* = .045), and residual lesions (aOR 6.86; 95 %CI 1.49–31.56; *P* = .013) were associated with refractoriness or recurrence.

**Conclusions:**

Pulmonary resection is a reasonable treatment modality for patients with refractory NTM-PD or major complications such as massive hemoptysis. The potential risk factors associated with unfavorable outcomes included female sex, preoperative positive mycobacterial culture, and residual lesions after surgery.

**Supplementary Information:**

The online version contains supplementary material available at 10.1186/s12890-021-01679-0.

## Background

The burden of infection caused by nontuberculous mycobacteria (NTM), defined as mycobacteria other than *M. leprae* and *M. tuberculosis* complex, is increasing globally [1]. While NTM can involve organs such as the skin, soft tissue, and lymph nodes, it predominantly causes infection in the lungs. *M. avium* complex (MAC), which includes *M. avium* and *M. intracellulare*, is the most common cause of NTM pulmonary disease (NTM-PD). *M. abscessus* complex (MABC), to which the *M. abscessus* subspecies *abscessus* (*M. abscessus*), *M. abscessus* subspecies *massiliense* (*M. massiliense*), and *M. abscessus* subspecies *bolletii* belong, is the next-most common etiology in many countries, including South Korea [2].

Recent practice guidelines for NTM-PD recommend treatment initiation rather than “watchful waiting” for patients meeting the clinical, radiographic, and microbiological criteria for diagnosis, especially in the context of positive sputum acid-fast bacilli smears and/or the presence of cavitary lesions [3]. However, the success rate of antibiotic treatment is unsatisfactory, with rates of 60.0–65.7% for MAC [4, 5] and 34.0–45.6% for MABC [6, 7]. Consequently, surgical resection is considered a reasonable adjunctive therapy, especially for patients with refractory disease despite rigorous medical treatment, with large cavitary lesions expected to be less penetrable by antibiotics, or with severe disease-related complications such as massive hemoptysis [1, 3, 8].

While previous studies on the surgical outcomes of adjunctive surgery have provided meaningful guidance and insight, these findings varied considerably by institution. For example, major outcomes such as negative sputum culture conversion or postoperative complications ranged from 57 to 100% and 0–46%, respectively [9–20]. Conflicting results and lack of standardized guidelines provide little help for patients and clinicians alike regarding the decision to perform surgery.

To resolve this uncertainty, more evidence on surgical outcomes is needed. This study described the outcomes of patients with NTM-PD who underwent surgical resection at a tertiary referral center in South Korea and provided a detailed account of complications and risk factors associated with refractoriness or recurrence after surgery for NTM-PD.

## Methods

### Study design and patient selection

This retrospective cohort study analyzed the medical records of patients with NTM-PD who met the diagnostic criteria of the American Thoracic Society/European Respiratory Society/European Society of Clinical Microbiology and Infectious Diseases/Infectious Diseases Society of America guidelines [3] and underwent surgical resection as adjunctive therapy between January 1, 2006, and December 31, 2020, at Seoul National University Hospital in South Korea. For patients with multiple pulmonary resections, the analysis considered only the first resection. This study was approved by the Institutional Review Board of Seoul National University Hospital (IRB No. 2104-094-1211).

### Data collection

Baseline patient demographics at the time of surgery, including age, sex, body mass index (BMI), smoking history, and underlying disease were collected. Data on acid-fast bacilli smear, mycobacterial culture, mycobacterial species identification using 16 S rRNA and *rpo*B sequencing [21, 22], and drug susceptibility test (DST) results; antibiotic regimen; and treatment duration before and after surgery were also obtained. DSTs were performed at the Korean Institute of Tuberculosis using the broth microdilution method. Inducible resistance, defined as the susceptible minimum inhibitory concentration (MIC) values (≤ 2 mg/L) to clarithromycin for three days but resistant MIC values (≥ 8 mg/L) at a longer incubation period, was tested by extending the incubation with clarithromycin to 14 days [3, 23]. Preoperative chest computed tomography (CT) images were acquired and classified as ‘fibrocavitary’, ‘non-cavitary nodular bronchiectatic’, or ‘cavitary nodular bronchiectatic’ by two pulmonologists blinded to the clinical data. The extent of disease was also evaluated. The presence of postoperative residual lesions, defined by remaining nodular, cavitary opacities, or multifocal bronchiectasis consistent with radiologic findings of NTM-PD, was assessed by comparing CT images before and after surgery. Discrepancies were resolved through discussion. Data on preoperative pulmonary function test results, surgical procedure, immediate postoperative complications, and their management were also collected. We also reviewed the pathology reports of the surgical specimens.

### Treatment outcome assessment

We assessed treatment outcomes according to the definitions provided by the NTM-NET consensus statement [24] and recent studies [16–20]. Negative culture conversion was defined as three or more consecutive negative mycobacterial cultures from respiratory samples collected over at least 3 months. Patients who did not achieve negative culture conversion after surgery were defined as refractory cases. Patients with sustained multiple consecutive negative mycobacterial cultures without positive cultures until the end of follow-up were considered microbiologically cured. Recurrence was defined as the re-emergence of at least two positive mycobacterial cultures from respiratory specimens following negative culture conversion after surgery.

### Statistical analysis

Categorical variables were reported as frequencies (percentages), while continuous variables were summarized as medians with interquartile ranges (IQRs). Patient groups were compared using Pearson’s chi-square or Fisher’s exact tests for categorical variables and Mann–Whitney U tests for continuous variables. Univariate logistic regression analysis was performed to identify the potential risk factors associated with refractoriness or recurrence. For multivariate logistic regression analysis, variables with statistical significance of *P* < .2 on univariate analysis were included, along with variables selected *a priori* based on the background knowledge of previous studies. *P* < .05 was considered statistically significant. Statistical analyses were conducted using R software (version 4.0.4, R Foundation for Statistical Computing, Vienna, Austria) using the *moonBook* package for descriptive analysis and fitting generalized linear models for logistic regression modeling.

## Results

### Patient characteristics

During the study period, 67 patients with NTM-PD (female 53 [79.1%]; male 14 [20.9%]) underwent surgical resection and were included in the study. The baseline characteristics of the patients at the time of surgery are summarized in Table 1. The median age and BMI were 57 years (IQR 50–65 years) and 20.1 kg/m^2^ (IQR 18.7–21.3 kg/m^2^), respectively. Fifty-nine (88.1%) patients had never smoked cigarettes, and 10 (14.9%) patients had a previous history of pulmonary tuberculosis.

**Table.1:** Baseline characteristics of 67 patients who underwent surgical resection

Characteristics	n = 67
Age years, median (IQR)	57 (50, 65)
Female sex, n (%)	53 (79.1)
BMI kg/m^2^ (n = 47), median (IQR)	20.1 (18.7, 21.3)
Smoking status, Never/former, n (%)	59 (88.1)/8 (11.9)
Underlying disease	
History of pulmonary TB, n (%)	10 (14.9)
Lung cancer, n (%)	2 (3)
Asthma, n (%)	2 (3)
COPD, n (%)	1 (1.5)
Other malignancy, n (%)	5 (7.5)
Diabetes mellitus, n (%)	3 (4.5)
Charlson comorbidity index, median (IQR)	2 (1, 3)
Causative *Mycobacterium* species	
MAC, n (%)	39 (58.2)
*M. avium*, n (%)	18 (26.9)
*M. intracellulare*, n (%)	21 (31.3)
MABC, n (%)	24 (35.8)
*M. abscessus*, n (%)	16 (23.9)
*M. massiliense*, n (%)	8 (11.9)
*M. kansasii*, n (%)	3 (4.5)
*M. fortuitum*, n (%)	1 (1.5)
Clarithromycin resistance in MABC	
*M. abscessus* (n = 13), S/IR or R, n (%)	2 (3)/11 (16.4)
*M. massiliense* (n = 8), S/IR or R, n (%)	7 (87.5)/1 (12.5)
Preoperative positive NTM culture, n (%)	54 (80.6)
CT pattern	
Non-cavitary nodular bronchiectatic, n (%)	19 (28.4)
Cavitary nodular bronchiectatic, n (%)	34 (50.7)
Fibrocavitary, n (%)	14 (20.9)
Disease extent	
Involving a single lobe, n (%)	7 (10.4)
Involving more than three lobes, n (%)	46 (68.7)
Bilateral involvement, n (%)	49 (73.1)
Involving all lobes, n (%)	12 (17.9)
Preoperative PFT	
FEV1/FVC ratio, median (IQR)	76.5 (70, 80.8)
% predicted FEV1, median (IQR)	94 (83.3, 101.8)
% predicted FVC, median (IQR)	90 (81, 99)
% DLCO, median (IQR)	87 (77, 96.5)
Preoperative NTM treatment	
On antibiotics treatment prior to surgery, n (%)	61 (91)
Guideline-adhering regimens, n (%)	58 (95.1)
Macrolide-based regimen with intravenous drugs, n (%)	29 (47.5)
Macrolide-based regimen without intravenous drugs, n (%)	31 (50.8)
Non-macrolide-based regimen, n (%)	1 (1.6)
Treatment duration, months, median (IQR)	14 (9, 25)
No antibiotic treatment before surgery, n (%)	6 (9)
Surgical indication	
Persistent NTM culture positivity, n (%)	54 (80.6)
Radiographic aggravation and/or persistent cavity, n (%)	7 (10.4)
Massive hemoptysis, n (%)	3 (4.5)
Initiation of therapy, n (%)	1 (1.5)
Others, n (%)	2 (3)

*IQR* Interquartile range, *BMI* body mass index, *TB* tuberculosis, *COPD* Chronic obstructive pulmonary disease, *MAC* *Mycobacterium avium* complex, *MABC* *Mycobacterium abscessus* complex, *S* susceptible, *IR* inducible resistance, *R* resistance, *NTM* nontuberculous mycobacteria, *CT* computed tomography, *PFT* pulmonary function test, *FEV1* forced expiratory volume in 1 s, *FVC* forced vital capacity, *DLCO* diffusing capacity of the lungs for carbon monoxide

Thirty-nine patients (58.2%) were positive for MAC (*M. avium*, 18 [26.9%]; *M. intracellulare*, 21 [31.3%]), while 24 (35.8%) patients were positive for MABC (*M. abscessus*, 16 [23.9%]; *M. massiliense*, 8 [11.9%]). Detailed characteristics, surgical indications, and types of procedures stratified by the type of NTM species are presented in Additional file 1: Table 1. Among the 61 patients whose DSTs were available, 11 (84.6%) of 13 patients infected with *M. abscessus* and one (12.5%) of eight patients infected with *M. massiliense* showed resistance or inducible resistance to clarithromycin, respectively. The most prevalent CT pattern in the study group was cavitary nodular bronchiectasis (34; 50.7%), followed by non-cavitary nodular bronchiectasis (19; 28.4%) and fibrocavitary bronchiectasis (14; 20.9%). Forty-nine (73.1%) patients showed bilateral involvement of the disease, while seven (10.4%) patients showed single-lobe involvement.

Before surgery, most patients (61; 91%) received antibiotic treatment for NTM-PD and among them, 58 patients (95.1%) were treated with guideline-adhering regimens: a combination of clarithromycin or azithromycin, rifampin, and ethambutol with consideration of amikacin or streptomycin for MAC; a combination of isoniazid, rifampin, and ethambutol for *M. kansasii*; and multidrug regimens including macrolides, amikacin, imipenem, or cefoxitin for MABC [1]. The median duration of antibiotic treatment before surgery was 14 months (IQR 9–25 months).

### Indications and types of surgical resection

The main indication for surgery was persistent positive mycobacterial culture (54; 80.6%) followed by longstanding cavity or radiographic aggravation (7; 10.4%) despite rigorous antibiotic treatment. Three patients underwent resection due to massive hemoptysis (4.5%). Another patient (1.5%) had his right middle lobe, the only region destroyed by *M. abscessus*, removed as initiation of NTM therapy. The remaining two patients (4.5%) underwent resection of a solitary nodule or cavitary lesion for diagnostic purposes. Fifty-eight patients (86.6%) underwent video-assisted thoracoscopic surgery (VATS); the others underwent open thoracotomy. The surgical procedures included pneumonectomy (4; 6.0%), bilobectomy with wedge resection (1; 1.5%), bilobectomy without segmentectomy or wedge resection (1; 1.5%), lobectomy with segmentectomy or wedge resection (15; 22.4%), lobectomy without segmentectomy or wedge resection (18; 26.9%), segmentectomy with wedge resection (11; 16.4%), segmentectomy without wedge resection (5; 7.5%), and wedge resection (12; 17.9%) (Table 2). The median length of hospital stay for surgery was 6 days (IQR 4–9 days).

**Table.2:** Types of procedures

Type of procedure	No. (%)
Pneumonectomy	4 (6)
Left	3 (4.5)
Right	1 (1.5)
Bilobectomy with wedge resection	1 (1.5)
Bilobectomy without wedge resection	1 (1.5)
Lobectomy with segmentectomy or wedge resection	15 (22.4)
Lobectomy without segmentectomy or wedge resection	18 (26.9)
Segmentectomy with segmentectomy or wedge resection	11 (16.4)
Segmentectomy without segmentectomy or wedge resection	5 (7.5)
Wedge resection with or without wedge resection	12 (17.9)
Video-assisted thoracoscopic surgery	58 (86.6)
Open thoracotomy	9 (13.4)

### Postoperative complications

The postoperative complications are summarized in Table 3. Nine patients experienced immediate postoperative complications, more commonly those who underwent open thoracotomy (five following open thoracotomy and four following VATS; *P* = .001). The most common postoperative complication was prolonged air leakage (3; 33.3%). Wound dehiscence requiring surgical closure occurred in patients who underwent VATS. One patient experienced delayed surgical site bleeding, which was successfully managed by hemoclipping. Only one patient in our study developed a bronchopleural fistula.

**Table.3:** Surgical complications and management

No.	Complication	Age	Sex	*Mycobacterial* species	Type of procedure	Thoracotomy	LOS, days	Management	Outcome
1	Prolonged air leakage	72	M	*M. avium*	RUL posterior and RLL superior bisegmentectomy	Yes	19	Pleurodesis	Controlled
2	Wound dehiscence	65	F	*M. abscessus* subsp. *abscessus*	RUL lobectomy	No	9	Primary wound closure	Controlled
3	Prolonged air leakage	73	F	*M. intracellulare*	RUL lobectomy with RLL wedge resection	Yes	34	Pleurodesis	Controlled
4	Postoperative delayed bleeding	45	F	*M. abscessus* subsp. *abscessus*	RUL wedge resection	No	8	Hemoclipping	Controlled
5	Pneumonia with parapneumonic effusion	68	M	*M. avium*	RUL lobectomy	Yes	21	Antibiotics	Controlled
6	Bronchopleural fistula, atrial fibrillation	84	M	*M. abscessus* subsp. *abscessus*	Lt. pneumonectomy	Yes	11	Closed tube thoracostomy, antibiotics	Controlled
7	Pneumonia	62	M	*M. intracellulare*	Lt. pneumonectomy	Yes	44	Antibiotics	Controlled
8	Wound dehiscence	46	F	*M. intracellulare*	RLL lobectomy	No	7	Primary wound closure	Controlled
9	Prolonged air leakage	60	M	*M. abscessus* subsp. *abscessus*	RML lobectomy	No	16	Pleurodesis, RUL wedge resection	Controlled

*LOS* Length of stay, *RUL* right upper lobe, *RLL* right lower lobe, *subsp* subspecies, *Lt* left, *RML* right middle lobe

### Treatment outcomes

**Fig. 1:**
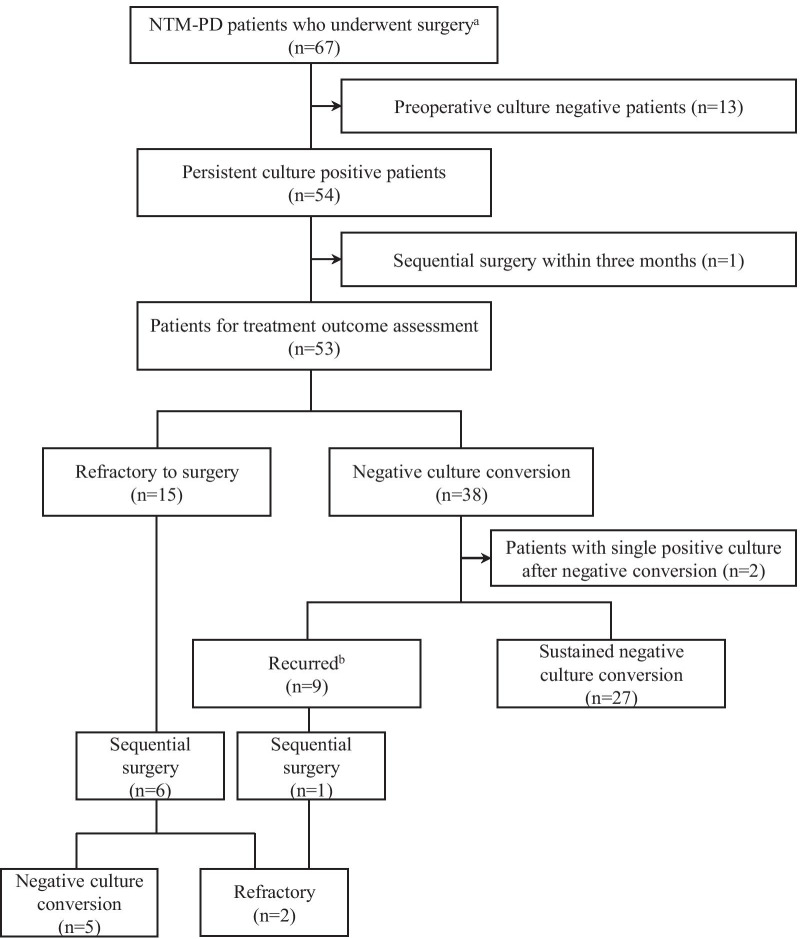
Treatment outcomes of 67 patients based on mycobacterial culture results. ^a^Between 1 January 2006 and 31 December 2020. ^b^Re-emergence of at least two positive mycobacterial cultures from respiratory specimen following negative culture conversion after surgery

Fifty-nine (88.1%) patients were administered antibiotics postoperatively for a median of 17.5 months (IQR 10.25–23.75 months). Of the 54 patients with persistent positive mycobacterial culture before surgery (one was omitted due to planned sequential operation within 3 months), 38 (71.7%) achieved initial negative culture conversion postoperatively. Among them, 27 (50.9%) patients maintained negative culture conversion throughout the median follow-up period of 38 months (IQR 17.5–59.5 months) while 9 (17.0%) experienced recurrence. Fifteen (28.3%) patients displayed persistently positive mycobacterial cultures and were defined as refractory cases. The details of the treatment outcomes are summarized in Fig. 1. One patient with recurrence and six refractory patients underwent sequential surgery, five of whom subsequently achieved negative culture conversion. The factors associated with the additional surgeries are summarized in Additional file 1: Table 2. All three patients whose indications for surgery were massive hemoptysis despite bronchial artery embolization remained stable without hemoptysis for a median of 14 months (range, 10–90 months), and none underwent additional intervention. None of the patients died within 90 days. The number of NTM-PD-related hospital admissions before and after surgery was 17.1 and 22.8 admissions per 100 person-years, respectively (*P* = .119). The number of emergency room (ER) visits before and after surgery was 20.7 and 18.3 visits per 100 person-years, respectively (*P* = .544). The changes in the type of surgical procedure and outcomes according to the time periods are summarized in Additional file 1: Table 3.

### Concomitant fungal infections

Five of the 67 patients had an account of numerous hyphae, suggestive of aspergillosis. The patient ages ranged from 47 to 69 years, and only one patient was male. None of the patients had underlying disease and four (80%) had complained of hemoptysis before surgery. The causative mycobacterial species included *M. avium* in two patients and *M. intracellulare*, *M. abscessus*, and *M. massiliense* in one patient each. Three patients had cavitary lesions; the other two showed extensive bronchiectasis and parenchymal destruction. Only one patient’s surgical specimen exhibited fungal organisms infiltrating the surrounding lung parenchyma, consistent with invasive pulmonary aspergillosis (IPA). This patient was consequently treated with itraconazole for 1 year, while others were closely followed without the administration of any antifungal agent. Two patients were tested for *Aspergillus* immunoglobulin G, and all the test results were positive. Fungal organisms were not isolated from the sputum cultures studied in two other patients.

### Risk Factors associated with refractoriness or recurrence

A total of 15 refractory and 12 recurrent patients were grouped for logistic regression analysis. This analysis indicated postoperative residual lesions (odds ratio [OR] 4.74, 95% confidence interval [CI], 1.32–16.96, *P* = .017) as a potential predictor of refractoriness or recurrence after surgery. Multivariate analysis revealed that female sex (adjusted OR, 6.63; 95% CI, 1.04–42.4; *P* = .046), preoperative positive mycobacterial culture (adjusted OR, 5.87; 95% CI, 1.04–33.08, *P* = .045), and residual lesions after surgery (adjusted OR, 6.86; 95% CI, 1.49–31.56; P = .013) increased the odds of refractoriness or recurrence, while age, BMI, and type of surgical procedure were not associated with treatment outcomes (Table 4). Predictors of treatment outcomes by species type are presented in Additional file 1: Tables 4 and 5.

**Table.4:** Risk factors associated with NTM-PD refractoriness or recurrence after surgery

Characteristics	OR (95 %CI)	*P*-value	aOR (95 %CI)	*P*-value
Age	1.03 (0.98–1.08)	0.314	1.03 (0.96–1.11)	0.350
Female sex	2.78 (0.66–11.80)	0.165	6.63 (1.04–42.4)	0.046
BMI kg m^− 2^	0.83 (0.65–1.07)	0.150	0.77 (0.58–1.03)	0.079
Causative *Mycobacterium* species		> 0.999		
*Mycobacterium avium* complex	Ref			
*Mycobacterium abscessus* complex	1 (0.33–3.02)			
Preoperative positive NTM culture	2.78 (0.66–11.80)	0.165	5.87 (1.04–33.08)	0.045
Surgical procedure and complications				
Thoracotomy	1.17 (0.26–5.23)	0.833		
Pneumonectomy	2.40 (0.21–28.05)	0.485	1.80 (0.10–32.59)	0.692
Complications	2.12 (0.46–9.86)	0.337		
Postoperative radiologic findings				
Residual lesions	4.74 (1.32–16.96)	0.017	6.86 (1.49–31.56)	0.013
Residual cavity	2.36 (0.61–9.18)	0.215		

*OR* odds ratio, *CI* confidence interval, *aOR* adjusted odds ratio, *BMI* body mass index, *NTM* nontuberculous mycobacteria

## Discussion

This study investigated the outcomes of adjunctive surgical treatment in 67 patients with NTM-PD and derived the risk factors associated with unfavorable outcomes. Following surgical resection, 70% of the patients who were refractory to prior antibiotic treatment achieved initial negative culture conversion. After achieving culture conversion, half of these patients remained culture-negative during a median follow-up of 38 months. In addition to mycobacterial eradication, surgical resection also resolved uncontrolled hemoptysis. Although approximately 13% of patients experienced postoperative complications, all of them recovered with appropriate measures and none died due to surgical resection. However, 15 patients were non-responsive to surgical resection. Unfavorable outcomes were associated with female sex, preoperative positive mycobacterial culture, and residual lesions after surgical resection. While these are acceptable outcomes, the culture conversion rate in our study was lower than those reported previously [9–13, 15–20, 25, 26]. This may be explained by different patient compositions and outcome definitions across studies. For instance, while many studies did not include or had few patients with MABC infections, which are difficult to treat and resistant to many antibiotics, one-third of our study population was positive for MABC infection. Moreover, unlike other studies, we took a more conservative approach of assessing ‘true’ postoperative negative culture conversion among those who showed persistent positive mycobacterial cultures before surgery.

Our study results indicated that surgical resection in patients with NTM-PD can be performed safely without long-lasting morbidities. Although postoperative complications occurred in 13.4% of patients, all were managed without enduring morbidity. Only one patient required an additional surgical procedure, while the others recovered with conservative management. A larger proportion (86.6%) of patients who received VATS in the present study compared to those in other studies [9, 11, 12, 17, 18, 20, 27] may have improved the postoperative complication rate in our study. The postoperative complication rate was the lowest when most patients were treated with VATS (Additional file 1: Table 3).

Chronic pulmonary aspergillosis (CPA) following NTM-PD, which is caused by *Aspergillus* species, has been increasingly reported. The prevalence of CPA in NTM-PD ranges from 3.9 to 16.7% [28, 29]. The risk factors associated with concomitant CPA in NTM-PD include the presence of fibrocavitary lesions or emphysema and the use of corticosteroids [29]. In our study, five patients had pathologic findings suggestive of CPA, one of whom was treated with an antifungal agent. This result emphasizes the importance of an awareness of combined fungal infections when treating NTM-PD.

In this study, female sex, preoperative positive mycobacterial culture, and postoperative residual lesions were associated with NTM-PD refractoriness or recurrence after surgery. The unfavorable outcomes in female patients could be explained in terms of female predilection for NTM-PD [31, 32], which might lead to higher recurrence rates in female patients. In this study, all of the recurrent cases were women. This predilection could be caused by genetic or hormonal differences [31, 32]. Interestingly, previously reported risk factors such as old age, longer period from initial medical treatment to surgery, and infection by non-*M. avium* species were not predictors of outcome in the present study. This may be due to differences in the size and composition of the study population.

Similar to previous studies, we confirmed the importance of residual lesions after surgery [17, 33, 34]. The most common radiographic feature of residual lesions was non-cavitary nodular bronchiectatic (69.6%), followed by cavitary nodular bronchiectatic (21.7%) and fibrocavitary pattern (2.2%). Overwhelmingly many patients (93.5%) with residual lesions had bilateral lung involvement before surgery. Most patients in our study underwent surgery in order to minimize the mycobacterial burden in otherwise palliative setting. Thus, we adopted limited resection strategy to conserve pulmonary function. But, as Yamada and colleagues suggested [34] and our result underscores, extensive resection that minimizes residual lesion may be required for proper disease control. As Togo and colleagues emphasized [33], more study regarding acceptable extent of remnant lesions after surgery may be necessary.

Our study has several limitations. This retrospective cohort study was conducted in a single institution. A relatively small number of patients were identified over 15 years, which might imply a selection bias, where only tolerable patients with few comorbidities and good functional status were selected for surgery. Treatment outcomes were evaluated according to a widely used operational definition, which is mainly based on expert consensus. Thus, cautious interpretation of the results is advised. The changes in symptoms or quality of life following surgery could not be quantitatively measured. However, we have provided the changes in the number of NTM-PD-related hospital admissions and ER visits as surrogate outcomes. Well-designed prospective randomized control trials comparing the outcomes of antibiotic-only treatment and adjunctive surgery are required. Yet, our study included a relatively large number of patients with MABC compared to other studies. Moreover, most of the patients in our institution were treated with VATS, which depicts a more realistic and updated picture of surgical outcomes of patients with NTM-PD.

## Conclusions

In conclusion, pulmonary resection can be a valuable treatment option for patients with NTM-PD who are refractory to antibiotic treatment or experience disease-related complications such as hemoptysis. The potential factors associated with unfavorable outcomes included female sex, preoperative positive mycobacterial culture, and residual lesions after surgery. Postoperative complications can be managed with low morbidity and mortality in experienced institutions.

## Supplementary Information

**Additional file 1**. Baseline characteristics and risk factors associated with unfavorable outcomes by NTM species & risk factors associated with additional surgery.

## Data Availability

The dataset used are available from the corresponding author on reasonable request.

## Abbreviations

aOR: Adjusted odds ratio
BMI: Body mass index
CPA: Chronic pulmonary aspergillosis
CT: Computed tomography
CI: Confidence interval
DST: Drug susceptibility test
ER: Emergency room
IQR: Interquartile range
IPA: Invasive pulmonary aspergillosis
MABC: *Mycobacterium abscessus* complex
MAC: *Mycobacterium avium* complex
MIC: Minimum inhibitory concentration
NTM: Nontuberculous mycobacteria
NTM-PD: Nontuberculous mycobacterial pulmonary disease
VATS: Video-assisted thoracoscopic surgery

## References

[1] Griffith DE, Aksamit T, Brown-Elliott BA (2007). An official ATS/IDSA statement: diagnosis, treatment, and prevention of nontuberculous mycobacterial diseases. Am J Respir Crit Care Med.

[2] Ko RE, Moon SM, Ahn S (2018). Changing epidemiology of nontuberculous mycobacterial lung diseases in a tertiary referral hospital in Korea between 2001 and 2015. J Korean Med Sci.

[3] Daley CL, Iaccarino JM, Lange C (2020). Treatment of nontuberculous mycobacterial pulmonary disease: An official ATS/ERS/ESCMID/IDSA clinical practice guideline. Clin Infect Dis.

[4] Kwak N, Park J, Kim E, Lee CH, Han SK, Yim JJ (2017). Treatment outcomes of mycobacterium avium complex lung disease: A systematic review and meta-analysis. Clin Infect Dis.

[5] Diel R, Nienhaus A, Ringshausen FC (2018). Microbiologic outcome of interventions against mycobacterium avium complex pulmonary disease: A systematic review. Chest.

[6] Kwak N, Dalcolmo MP, Daley CL (2019). Mycobacterium abscessus pulmonary disease: Individual patient data meta-analysis. Eur Respir J.

[7] Diel R, Ringshausen F, Richter E, Welker L, Schmitz J, Nienhaus A (2017). Microbiological and clinical outcomes of treating non-mycobacterium avium complex nontuberculous mycobacterial pulmonary disease: A systematic review and meta-analysis. Chest.

[8] Mitchell JD (2019). Surgical treatment of pulmonary nontuberculous mycobacterial infections. Thorac Surg Clin.

[9] Nelson KG, Griffith DE, Brown BA, Wallace RJ (1998). Jr. Results of operation in mycobacterium avium-intracellulare lung disease. Ann Thorac Surg.

[10] Shiraishi Y, Fukushima K, Komatsu H, Kurashima A (1998). Early pulmonary resection for localized mycobacterium avium complex disease. Ann Thorac Surg.

[11] Shiraishi Y, Nakajima Y, Takasuna K, Hanaoka T, Katsuragi N, Konno H (2002). Surgery for mycobacterium avium complex lung disease in the clarithromycin era. Eur J Cardiothorac Surg.

[12] Sherwood JT, Mitchell JD, Pomerantz M (2005). Completion pneumonectomy for chronic mycobacterial disease. J Thorac Cardiovasc Surg.

[13] Watanabe M, Hasegawa N, Ishizaka A (2006). Early pulmonary resection for mycobacterium avium complex lung disease treated with macrolides and quinolones. Ann Thorac Surg.

[14] Jarand J, Levin A, Zhang L, Huitt G, Mitchell JD, Daley CL (2011). Clinical and microbiologic outcomes in patients receiving treatment for mycobacterium abscessus pulmonary disease. Clin Infect Dis.

[15] Shiraishi Y, Katsuragi N, Kita H, Hyogotani A, Saito MH, Shimoda K (2013). Adjuvant surgical treatment of nontuberculous mycobacterial lung disease. Ann Thorac Surg.

[16] Kang HK, Park HY, Kim D (2015). Treatment outcomes of adjuvant resectional surgery for nontuberculous mycobacterial lung disease. BMC Infect Dis.

[17] Asakura T, Hayakawa N, Hasegawa N (2017). Long-term outcome of pulmonary resection for nontuberculous mycobacterial pulmonary disease. Clin Infect Dis.

[18] Aznar ML, Zubrinic M, Siemienowicz M (2018). Adjuvant lung resection in the management of nontuberculous mycobacterial lung infection: A retrospective matched cohort study. Respir Med.

[19] Yamada K, Seki Y, Nakagawa T, Hayashi Y, Yagi M, Ogawa K (2019). Outcomes and risk factors after adjuvant surgical treatments for mycobacterium avium complex lung disease. Gen Thorac Cardiovasc Surg.

[20] Fukushima K, Miki M, Matsumoto Y (2020). The impact of adjuvant surgical treatment of nontuberculous mycobacterial pulmonary disease on prognosis and outcome. Respir Res.

[21] Kim BJ, Lee SH, Lyu MA (1999). Identification of mycobacterial species by comparative sequence analysis of the RNA polymerase gene (rpoB). J Clin Microbiol.

[22] Ben Salah I, Adekambi T, Raoult D, Drancourt M (2008). Rpob sequence-based identification of mycobacterium avium complex species. Microbiology (Reading).

[23] Nash KA, Brown-Elliott BA, Wallace RJ (2009). A novel gene, erm(41), confers inducible macrolide resistance to clinical isolates of Mycobacterium abscessus but is absent from Mycobacterium chelonae. Antimicrob Agents Chemother.

[24] van Ingen J, Aksamit T, Andrejak C (2018). Treatment outcome definitions in nontuberculous mycobacterial pulmonary disease: An ntm-net consensus statement. Eur Respir J.

[25] Shiraishi Y, Nakajima Y, Katsuragi N, Kurai M, Takahashi N (2004). Pneumonectomy for nontuberculous mycobacterial infections. Ann Thorac Surg.

[26] van Ingen J, Verhagen AF, Dekhuijzen PN (2010). Surgical treatment of non-tuberculous mycobacterial lung disease: Strike in time. Int J Tuberc Lung Dis.

[27] Yotsumoto T, Inoue Y, Fukami T, Matsui H (2020). Pulmonary resection for nontuberculous mycobacterial pulmonary disease: Outcomes and risk factors for recurrence. Gen Thorac Cardiovasc Surg.

[28] Ishikawa S, Yano S, Kadowaki T (2011). [clinical analysis of non-tuberculous mycobacteriosis cases complicated with pulmonary aspergillosis]. Kekkaku.

[29] Zoumot Z, Boutou AK, Gill SS (2014). Mycobacterium avium complex infection in non-cystic fibrosis bronchiectasis. Respirology.

[30] Phoompoung P, Chayakulkeeree M. Chronic pulmonary aspergillosis following nontuberculous mycobacterial infections: an emerging disease. J Fungi (Basel). 2020;6(4). 10.3390/jof6040346 PMC7762599 33302348

[31] Chan ED, Iseman MD (2010). Slender, older women appear to be more susceptible to nontuberculous mycobacterial lung disease. Gend Med.

[32] Mirsaeidi M, Sadikot RT (2015). Gender susceptibility to mycobacterial infections in patients with non-cf bronchiectasis. Int J Mycobacteriol.

[33] Togo T, Atsumi J, Hiramatsu M (2020). Residual destructive lesions and surgical outcome in Mycobacterium avium complex pulmonary disease. Ann Thorac Surg.

[34] Yamada K, Seki Y, Nakagawa T (2021). Extensive lung resection for nontuberculous mycobacterial lung disease with multilobar lesions. Ann Thorac Surg.

