# Supplementary material for: Outcomes of adjunctive surgery for nontuberculous mycobacterial pulmonary disease
Source: BMC Pulm Med. 2021 Oct 6;21:312. doi: 10.1186/s12890-021-01679-0 (PMC8496107; doi:10.1186/s12890-021-01679-0)
Supplement: Supplementary file 1 — Additional file 1. Baseline characteristics and risk factors associated with unfavorable outcomes by NTM species & risk factors associated with additional surgery. [file 12890_2021_1679_MOESM1_ESM.docx]

**Supplementary Table 1. Characteristics, surgical indications, and types of procedure by NTM species**

| Characteristics | MAC (n=39) | MABC (n=24) | Others (n=4) | *P* value |
| --- | --- | --- | --- | --- |
| **Age** years, median (IQR) | 60.0 (50.0, 66.0) | 56.5 (50.0, 61.0) | 45.0 (41.0, 50.5) | 0.039 |
| **Female** sex, n (%) | 31 (79.5) | 19 (79.2) | 3 (75.0) | 0.978 |
| **BMI** kg•m^-2^, median (IQR) | 19.9 (18.5, 21.3) | 20.3 (19.3, 21.4) | 20.4 (20.0, 21.9) | 0.444 |
| **Smoking status**, Never / Former, n (%) | 31 (79.5) / 5 (12.8) | 19 (79.2) / 2 (8.3) | 3 (75.0) / 1 (25.0) | 0.614 |
| **Underlying disease** |  |  |  |  |
| History of TB, n (%) | 5 (12.8) | 5 (20.8) | 0 (0.0) | 0.473 |
| Lung cancer, n (%) | 2 (5.1) | 0 (0.0) | 0 (0.0) | 0.477 |
| Asthma, n (%) | 2 (5.1) | 0 (0.0) | 0 (0.0) | 0.477 |
| COPD, n (%) | 1 (2.6) | 0 (0.0) | 0 (0.0) | 0.695 |
| Other malignancy, n (%) | 4 (10.2) | 0 (0.0) | 1 (25.0) | 0.125 |
| Diabetes mellitus, n (%) | 2 (5.1) | 1 (4.2) | 0 (0.0) | 0.891 |
| Charlson comorbidity index, median (IQR) | 2.0 (1.0, 3.0) | 2.0 (1.0, 2.0) | 1.5 (0.5, 2.0) | 0.191 |
| **Preoperative sputum examination within 1 month** | |  |  |  |
| Positive acid fast smear, n (%) | 13 (33.3) | 4 (16.7) | 0 (0.0) | 0.163 |
| Positive NTM culture, n (%) | 32 (82.1) | 19 (79.2) | 3 (75.0) | 0.921 |
| **CT pattern** |  |  |  |  |
| Fibrocavitary, n (%) | 9 (23.1) | 4 (16.7) | 1 (25.0) | 0.814 |
| Non-cavitary nodular bronchiectatic, n (%) | 9 (23.1) | 9 (37.5) | 1 (25.0) | 0.462 |
| Cavitary nodular bronchiectatic, n (%) | 21 (53.8) | 11 (45.8) | 2 (50.0) | 0.826 |
| **Disease extent** |  |  |  |  |
| Bilateral involvement, n (%) | 32 (82.1) | 15 (62.5) | 2 (50.0) | 0.132 |
| More than three lobes, n (%) | 30 (76.9) | 15 (62.5) | 1 (25.0) | 0.074 |
| Involved all lobes, n (%) | 7 (17.9) | 5 (20.8) | 0 (0.0) | 0.603 |
| Confied to single lobe, n (%) | 5 (12.8) | 0 (0.0) | 2 (50.0) | 0.008 |
| **Preoperative PFT** |  |  |  |  |
| FEV1/FVC ratio, median (IQR) | 74.0 (70.0, 80.0) | 77.0 (71.0, 79.5) | 83.0 (76.0, 89.5) | 0.268 |
| % FEV1, median (IQR) | 93.0 (82.5, 101.0) | 94.0 (83.5, 102.0) | 97.5 (93.5, 101.5) | 0.723 |
| % FVC, median (IQR) | 90.0 (81.0, 101.5) | 90.0 (82.5, 95.5) | 91.5 (82.0, 100.0) | 0.644 |
| % DLCO, median (IQR) | 87.0 (76.0, 97.5) | 86.0 (81.0, 93.0) | 88.0 (82.0, 100.0) | 0.931 |
| **Preoperative NTM treatment** | |  |  |  |
| On antibiotics treatment prior to surgery, n (%) | 37 (94.9) | 21 (87.5) | 3 (75.0) | 0.311 |
| Macrolide based regimen with intravenous drugs, n (%) | 13 (33.3) | 16 (66.7) | 0 (0.0) | 0.009 |
| Macrolide based regimen without intravenous drugs, n (%) | 24 (61.5) | 5 (20.8) | 2 (50.0) | 0.021 |
| Non-Macrolide based regimen, n (%) | 0 (0.0) | 0 (0.0) | 1 (25.0) | 0.001 |
| Treatment duration, months, median (IQR) | 14.0 (10.0, 27.0) | 12.0 (5.0, 20.0) | 20.0 (14.5, 21.5) | 0.117 |
| **Surgical indication** |  |  |  | 0.197 |
| Persistent NTM culture positivity, n (%) | 32 (82.1) | 19 (79.2) | 3 (75.0) |  |
| Radiographic aggravation and/or persistent cavity, n (%) | 5 (12.8) | 2 (8.3) | 0 (0.0) |  |
| Massive hemoptysis, n (%) | 1 (2.6) | 2 (8.3) | 0 (0.0) |  |
| Initiation of therapy, n (%) | 0 (0.0) | 1 (4.2) | 0 (0.0) |  |
| Others, n (%) | 1 (2.6) | 0 (0.0) | 1 (25.0) |  |
| **Types of procedure** |  |  |  | 0.901 |
| Pneumonectomy | 2 (5.1) | 2 (8.3) | 0 (0.0) |  |
| Left | 2 (5.1) | 1 (4.2) | 0 (0.0) |  |
| Right | 0 (0.0) | 1 (4.2) | 0 (0.0) |  |
| Bilobectomy with wedge resection | 0 (0.0) | 1 (4.2) | 0 (0.0) |  |
| Bilobectomy without wedge resection | 0 (0.0) | 1 (4.2) | 0 (0.0) |  |
| Lobectomy with segmentectomy or wedge resection | 9 (23.1) | 5 (20.8) | 1 (25.0) |  |
| Lobectomy without segmentectomy or wedge resection | 12 (30.8) | 5 (20.8) | 1 (25.0) |  |
| Segmentectomy with segmentectomy or wedge resection | 7 (17.9) | 4 (16.7) | 0 (0.0) |  |
| Segmentectomy without segmentectomy or wedge resection | 3 (7.7) | 1 (4.2) | 1 (25.0) |  |
| Wedge resection with or without wedge resection | 6 (15.4) | 5 (20.8) | 1 (25.0) |  |

Abbreviations: MAC, *Mycobacterium avium* complex; MABC, *Mycobacterium abscessus* complex; IQR, interquartile range; BMI, body mass index; TB, tuberculosis; COPD, chronic obstructive pulmonary disease; CT, computed tomography; PFT, pulmonary function test; FEV1, forced expiratory volume in 1 s; FVC, forced vital capacity; DLCO, diffusing capacity of the lungs for carbon monoxide; NTM, nontuberculous mycobacterium.

**Supplementary Table 2. Factors associated with additional surgery**

| Characteristics | OR (95% CI) | *P*-value | aOR (95% CI) | *P*-value |
| --- | --- | --- | --- | --- |
| **Age** | 0.98 (0.91-1.05) | 0.506 | 0.90 (0.81-1.00) | 0.060 |
| **Female** | 0.91 (0.17-4.97) | 0.916 |  |  |
| **BMI** | 0.83 (0.57-1.21) | 0.334 |  |  |
| **Former or current smoker** | 0.91 (0.10-8.42) | 0.934 |  |  |
| **Causative *Mycobacterium* species** |  |  |  |  |
| *Mycobacterium abscessus* complex | 3.05 (0.35-26.35) | 0.311 | 6.73 (0.61-74.56) | 0.120 |
| **Clarithromycin Resistance** |  |  |  |  |
| **Preoperative sputum examination within 1 month** |  |  |  |  |
| Positive acid fast smear | 0.82 (0.15-4.38) | 0.816 |  |  |
| Positive NTM culture | 2.09 (0.24-18.35) | 0.507 |  |  |
| **CT pattern** |  |  |  |  |
| Non-cavitary nodular bronchiectatic | 1.31 (0.29-5.89) | 0.723 |  |  |
| Cavitary nodular bronchiectatic | 2.14 (0.49-9.40) | 0.312 |  |  |
| **Disease extent** |  |  |  |  |
| Bilateral involvement | 3.32 (0.38-28.60) | 0.275 |  |  |
| **Preoperative NTM treatment** |  |  |  |  |
| Treatment duration | 1.01 (0.96-1.06) | 0.676 |  |  |
| **Surgical procedure and complication** |  |  |  |  |
| Complication | 2.08 (0.36-12.09) | 0.414 |  |  |
| **Postoperative radiologic findings** |  |  |  |  |
| Residual lesions | 4.21 (0.49-36.08) | 0.190 |  |  |
| Residual cavity | 3.12 (0.65-15.08) | 0.156 |  |  |

Abbreviations: aOR, adjusted odds ratio; BMI, body mass index; CT, computed tomography; NTM, nontuberculous mycobacterium

**Supplementary Table 3. Surgical procedure and outcome according to the time period**

| Characteristics | Surgery  before 2015 (n=13) | Surgery  between 2015 and 2017 (n=24) | Surgery  between 2018 and 2020 (n=30) | *P* value for trend^*^ |
| --- | --- | --- | --- | --- |
| **Surgical procedures** |  |  |  |  |
| Video-assisted thoracoscopic surgery | 9 (69.2) | 19 (79.2) | 30 (100) | 0.003 |
| Pneumonectomy | 0 (0) | 4 (16.7) | 0 (0) | 0.491 |
| Sublobar resection | 6 (46.2) | 10 (41.7) | 12 (40.0) | 0.719 |
| **Postoperative radiologic findings** |  |  |  |  |
| Residual lesions | 10 (76.9) | 16 (66.7) | 20 (66.7) | 0.562 |
| Residual cavity | 4 (30.8) | 3 (12.5) | 4 (13.3) | 0.226 |
| **Outcomes** |  |  |  |  |
| Post-operative complication | 2 (15.4) | 5 (20.8) | 2 (6.7) | 0.282 |
| Refractoriness | 3 (23.1) | 7 (29.2) | 5 (17.9) | 0.486 |
| Recurrence | 4 (40.0) | 6 (35.3) | 2 (10.0) | 0.034 |
| **Follow-up duration** | 7.2 (6.2, 8.4) | 3.9 (3.4, 4.6) | 1.1 (0.8, 2.2) | < 0.001 |

* Categorical and continuous variables were tested using the Cochran-Armitage and the Kendall rank correlation test for trend, respectively.

**Supplementary Table 4. Factors associated with refractoriness or recurrence after surgery in *Mycobacterium avium* complex**

| Characteristics | OR (95% CI) | *P*-value | aOR (95% CI) | *P*-value |
| --- | --- | --- | --- | --- |
| **Age** | 1.04 (0.97-1.11) | 0.307 | 1.09 (0.99-1.20) | 0.092 |
| **Female** | 2.88 (0.48-17.45) | 0.249 | 5.94 (0.65-54.55) | 0.116 |
| **BMI** | 0.90 (069-1.17) | 0.435 |  |  |
| **Preoperative sputum examination within 1 month** |  |  |  |  |
| Positive acid fast smear | 3.11 (0.72-13.44) | 0.129 |  |  |
| Positive NTM culture | 6.15 (0.64-59.46) | 0.116 | 22.01 (1.14-425.98) | 0.041 |
| **CT pattern** |  |  |  |  |
| Fibrocavitary | 0.43 (0.09-2.09) | 0.295 |  |  |
| Non-cavitary nodular bronchiectatic | 4.36 (0.74-25.74) | 0.104 |  |  |
| Cavitary nodular bronchiectatic | 0.71 (0.19-2.69) | 0.616 |  |  |
| **Disease extent** |  |  |  |  |
| Bilateral involvement | 1.33 (0.25-7.08) | 0.736 |  |  |
| Confied to single lobe | 0.22 (0.02-2.19) | 0.196 |  |  |
| **Preoperative NTM treatment** |  |  |  |  |
| On antibiotics treatment prior to surgery | 0.94 (0.05-16.35) | 0.967 |  |  |
| Treatment duration | 1.03 (0.98-1.09) | 0.266 |  |  |
| **Surgical procedure and complication** |  |  |  |  |
| Thoracotomy | 1.07 (0.18-6.22) | 0.939 |  |  |
| Complication | 1.71 (0.25-11.78) | 0.584 |  |  |
| **Postoperative radiologic findings** |  |  |  |  |
| Residual lesions | 3.75 (0.64-22.04) | 0.144 | 5.81 (0.70-48.54) | 0.104 |
| Residual cavity | 2.08 (0.41-10.53) | 0.375 |  |  |

Abbreviations: aOR, adjusted odds ratio; BMI, body mass index; NTM, nontuberculous mycobacterium; CT, computed tomography

**Supplementary Table 5. Factors associated with refractoriness^*^ after surgery in *Mycobacterium abscessus* complex**

| Characteristics | OR (95% CI) | *P*-value | aOR (95% CI) | *P*-value |
| --- | --- | --- | --- | --- |
| Age years | 0.96 (0.87-1.07) | 0.499 |  |  |
| Female sex | 0.91 (0.07-12.52) | 0.943 |  |  |
| BMI | 0.33 (0.11-1.03) | 0.057 | 0.14 (0.02-0.99) | 0.049 |
| **Preoperative sputum examination within 1 month** |  |  |  |  |
| Positive acid fast smear | 6.00 (0.42-85.25) | 0.186 | 38.45 (0.24-6292.31) | 0.161 |
| **CT pattern** |  |  |  |  |
| Fibrocavitary | 0.67 (0.05-8.16) | 0.751 |  |  |
| Non-cavitary nodular bronchiectatic | 1.12 (0.14-8.88) | 0.911 |  |  |
| Cavitary nodular bronchiectatic | 1.17 (0.17-8.09) | 0.876 |  |  |
| **Disease extent** |  |  |  |  |
| Bilateral involvement | 0.89 (0.11-7.02) | 0.911 |  |  |
| **Preoperative NTM treatment** |  |  |  |  |
| On antibiotics treatment prior to surgery | 0.91 (0.07-12.52) | 0.943 |  |  |
| Treatment duration | 0.96 (0.88-1.06) | 0.454 |  |  |
| **Surgical procedure and complication** |  |  |  |  |
| Complication | 6.00 (0.42-85.25) | 0.186 |  |  |
| **Postoperative radiologic findings** |  |  |  |  |
| Residual lesions | 0.89 (0.11-7.02) | 0.911 |  |  |
| Residual cavity | 1.10 (0.08-15.15) | 0.943 |  |  |

Abbreviations: aOR, adjusted odds ratio; BMI, body mass index; NTM, nontuberculous mycobacterium; CT, computed tomography

^*^Dependent variable was set as ‘refractoriness,’ defined as inability to achieve negative conversion after surgery.
